# An Absolutely Conserved Tryptophan in the Stem of the Envelope Protein E of Flaviviruses Is Essential for the Formation of Stable Particles

**DOI:** 10.3390/v13091727

**Published:** 2021-08-30

**Authors:** Iris Medits, Franz X. Heinz, Karin Stiasny

**Affiliations:** Center for Virology, Medical University of Vienna, 1090 Vienna, Austria; iris.medits@meduniwien.ac.at (I.M.); franz.x.heinz@meduniwien.ac.at (F.X.H.)

**Keywords:** flaviviruses, envelope protein E, stem region, flavivirus assembly, tick-borne encephalitis virus

## Abstract

The major envelope protein E of flaviviruses contains an ectodomain that is connected to the transmembrane domain by the so-called “stem” region. In mature flavivirus particles, the stem is composed of two or three mostly amphipathic α-helices and a conserved sequence element (CS) with an undefined role in the viral life cycle. A tryptophan is the only residue within this region which is not only conserved in all vector-borne flaviviruses, but also in the group with no known vector. We investigated the importance of this residue in different stages of the viral life cycle by a mutagenesis-based approach using tick-borne encephalitis virus (TBEV). Replacing W421 by alanine or histidine strongly reduced the release of infectious virions and their thermostability, whereas fusion-related entry functions and virus maturation were still intact. Serial passaging of the mutants led to the emergence of a same-site compensatory mutation to leucine that largely restored these properties of the wildtype. The conserved tryptophan in CS (or another big hydrophobic amino acid at the same position) is thus essential for the assembly and infectivity of flaviviruses by being part of a network required for conferring stability to infectious particles.

## 1. Introduction

**Figure 1:**
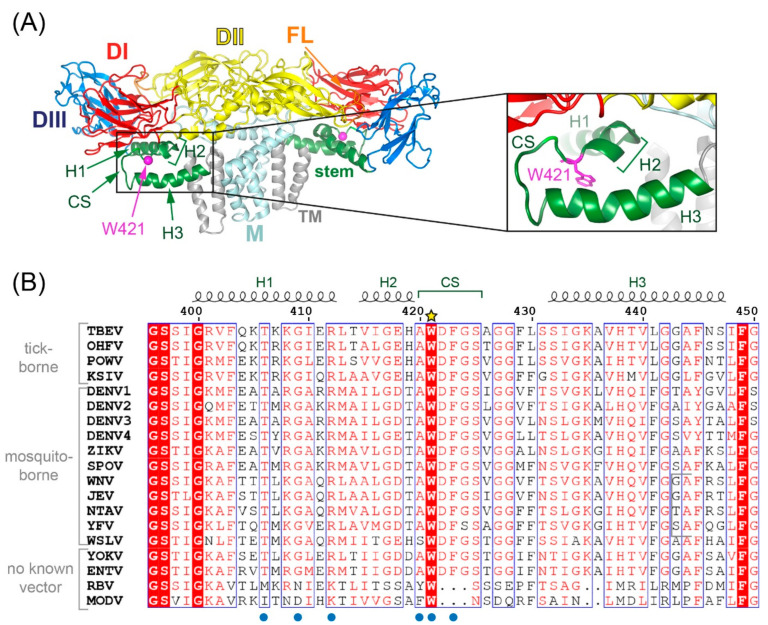
Structure of the TBEV (E-M)2 dimer on the surface of mature virions and an alignment of amino acid sequences of the stem. (**A**) Ribbon diagram of TBEV E and M proteins as a building block of mature virions (PDB 5o6a [9]). Color code: domain I (DI), red; domain II (DII), yellow; domain III (DIII), blue; fusion loop (FL), orange; stem, green; transmembrane (TM) domains, gray. Inset: Close-up of residue W421 in CS of the stem. The structures were generated with PyMol (https://pymol.org, accessed on 5 July 2021). (**B**) Sequence alignment of stem regions of different flaviviruses using MAFFT (https://www.ebi.ac.uk/Tools/msa/mafft, accessed on 5 July 2021) and ENDscript (http://espript.ibcp.fr/ESPript/ESPript/, accessed on 5 July 2021) [19]). The yellow star above the sequences labels the conserved tryptophan targeted in this study, the blue dots below the contact residues of E in the pocket site 1 with lipids [12]. TBEV, tick-borne encephalitis virus; OHFV, Omsk hemorrhagic virus; POWV, Powassan virus; KSIV, Karshi virus; DENV1–4, dengue virus serotypes 1–4; ZIKV, Zika virus; SPOV, Spondweni virus; WNV, West Nile virus; JEV, Japanese encephalitis virus; NTAV, Ntaya virus; YFV, yellow fever virus; WSLV, Wesselsbron virus; YOKV, Yokose virus; ENTV, Entebbe bat virus; RBV, Rio Bravo virus; MODV, Modoc virus. GenBank accession numbers are listed in Section 2.18.

Flaviviruses comprise a number of arthropod-borne human pathogens such as tick-borne encephalitis (TBE), yellow fever, Japanese encephalitis (JE), Zika and dengue viruses. In their mature form, these small enveloped viruses are completely covered by an icosahedral shell of homodimers of the major surface glycoprotein E [1]. The three-dimensional structure of E exhibits the same basic organization in all flaviviruses (Figure 1A), although the degree of amino acid identity across different flaviviruses is relatively low [2]. The E glycoprotein is an elongated molecule consisting of three external domains (DI, DII, DIII), connected to a double membrane anchor by the so-called stem. This structural element consists of two or three mostly amphipathic helices (H1, H2, H3) [3,4,5,6,7,8,9,10,11,12] and is located between the E-ectodomain and the viral membrane (Figure 1A). Two regions in E are highly conserved, the fusion loop (FL) at the tip of DII and the so-called conserved sequence in the stem (CS, amino acids 420–425 in TBEV, [13]) that forms a loop between H2 and H3 (Figure 1A). The FL is crucial for mediating fusion during virus entry after virus uptake by receptor-mediated endocytosis [14], but the role of CS is less clearly defined.

During the viral life cycle, E undergoes several conformational changes that are necessary for the processes of virus assembly, maturation and fusion during entry. Non-infectious immature particles with heterotrimeric spikes of E with a second membrane protein (prM–precursor of membrane protein M) are formed by budding into the endoplasmic reticulum (ER) [15,16]. Cleavage of prM into pr and M during exocytosis takes place in the acidic trans-Golgi-network by the cellular protease furin after reorganization of the immature spikes into E homodimers in a herringbone-like lattice, with the cleaved pr still remaining attached [17]. The presence of prM and pr in immature forms of the virus prevents premature intracellular fusion during viral exocytosis, and shedding of pr upon release of the virus particles from the infected cell generates fusion-competent and mature infectious viruses [16]. Upon virus entry into host cells via endocytosis, the low pH of the endosome triggers a massive structural reorganization of E that mediates membrane fusion and results in the conversion of E dimers into trimers [2,14]. The residues of the stem upstream of CS have been shown to stabilize the trimer during this process [13].

The only amino acid in the CS that is conserved not only across tick-borne and mosquito-borne flaviviruses but also across those with unknown vectors (no-known vector flaviviruses [18]) is residue W421 (TBE virus, TBEV, numbering, Figure 1B). Recent high-resolution structures of mature Zika, Spondweni and dengue viruses revealed pockets involving the stem of E that can accommodate lipids (pocket factors) [10,11,12]. These sites have been hypothesized as being crucial for stabilizing mature virus particles [10,11,12] and are located on either side of H1, with the CS being involved in the site that is closer to the viral membrane (site 1 as defined in reference [12], see also Figure 1B). In our work, we addressed the role of W421 more specifically and investigated its influence on virus formation and/or entry. For this purpose, we replaced this residue by alanine or histidine and analyzed the effect of these mutations on TBEV assembly, maturation, infectivity and fusion-related processes. In addition, we studied compensatory mutations capable of resuscitating lost functions, occurring during serial passaging experiments of mutated viruses.

Our study shows that W421 does not play an important role in viral fusion-related processes but is essential for virus assembly by establishing interactions that confer stability to infectious mature particles. As revealed by a resuscitating mutation in passaged virus mutants, these stabilizing interactions could also be provided by another large hydrophobic amino acid (leucine) at the same position.

## 2. Materials and Methods

### 2.1. Mutagenesis of cDNA Clones

Point mutations were introduced into the infectious cDNA clone based on the European subtype TBEV virus strain Neudoerfl (Gene bank accession number U27495). The pTNd/c plasmid contains the entire genomic cDNA (sequence 1–11,141 nt) in the pBR322 vector under the control of the T7 transcription promoter [20].

To insert the mutations W421A, W421H and W421L, restriction-free (rf) cloning (also referred to as overlap extension PCR cloning or ligation-independent cloning) was performed as described in [21]. In a first PCR, the pSV plasmid (for SVP production, see below) was the template for amplification of a DNA fragment corresponding to the stem region, using a pair of hybrid primers (Table 1). The resulting PCR product was purified by agarose gel electrophoresis and served as “megaprimers” for the second PCR with a methylated pTNd/c plasmid as template. The parental plasmids were degraded with the methylation-sensitive restriction enzyme DpnI (New England Biolabs) for two hours at 37 °C and 20 min at 80 °C [21]. The final product was transformed into 10-beta competent E. coli cells (New England Biolabs) according to the manufacturer′s protocol. After verification by sequence analysis according to Sanger [22], the plasmid DNA was amplified by transformation into electrocompetent HB101 cells (Gibco BRL) as recommended in the manufacturer´s protocol. Primer design was done with the online tool for the design of restriction-free cloning projects (http://www.rf-cloning.org/, accessed on 5 July 2021) [21]).

### 2.2. Mutagenesis of Recombinant Subviral Particles (SVPs)

Point mutations were introduced into the pSV-PE WT plasmid encoding the structural proteins prM and E of the European subtype TBEV virus strain Neudoerfl (Gene bank accession number U27495) under the control of the SV40 promotor [23]. The mutations leading to W421A, W421H and W421L were introduced by site-directed mutagenesis (site-directed mutagenesis system from Invitrogen) following the manufacturer’s instructions (Life technologies). The primers (Eurofins Genomics, Ebersberg, Germany) used for site-directed mutagenesis are listed in Table 2.

### 2.3. Production of SVPs

COS-1 cells (American Type Culture Collection ATCC no. CRL-1650) were grown in Dulbecco´s modified Eagle´s medium (DMEM) supplemented with 10% fetal calf serum (FCS) and 1% Penicillin-Streptomycin-Glutamine (all from Gibco) at 37 °C.

SVPs were produced essentially as described previously [23,24,25]. Briefly, COS-1 cells were electroporated with 5 µg DNA using the GenePulser from BioRad with the following settings: 1.5 kV; 25 μF. 20 to 22 h after electroporation the medium was replaced by DMEM (25 mM HEPES, Gibco) without FCS. 48 h post-electroporation, the supernatant was harvested and cleared by centrifugation (10,000 rpm; 30 min; 4 °C: Beckmann JA 14). The cell culture supernatants were subjected to ultracentrifugation (44,000 rpm, 2 h, 4 °C: Beckman Ti45 rotor) and the pellets, containing SVPs, were resuspended in TAN buffer pH 8.0 (50 mM triethanolamine, 10 mM NaCl).

### 2.4. In Vitro Transcription of RNA

In vitro transcription of wildtype (WT) and mutant RNAs from the cDNAs of infectious clones was carried out as described previously [26,27]. 6 µg cDNA were digested with the restriction enzyme NheI (Thermo Fisher Scientific, Waltham, MA, USA) and gaps were filled with the Klenow fragment (Thermo Fisher Scientific). After phenol-chloroform purification, RNA was synthesized using the T7 Megascript transcription kit (Ambion, Austin, TX, USA) followed by RNA purification with the RNeasy Clean up system (Qiagen, Hilden, Germany) according to the manufacturer´s protocol. The concentration of RNA was determined with the NanoDrop 2000 spectrophotometer (Thermo Fisher Scientific), and the quality of transcription was controlled by RNA gel electrophoresis.

### 2.5. Production of Virus Particles

Baby hamster kidney (BHK-21) cells (ATCC no. CCL-10) were grown in Eagle´s minimum essential medium (MEM, Sigma-Aldrich, St. Louis, MO, USA) supplemented with 5% FCS, 1% L-glutamine and 0.5% neomycin at 37 °C.

Cells at a density of 1.2 × 10^6^ /mL in Dulbecco´s phosphate buffered saline (DPBS) were electroporated with 7.8 µg in vitro transcribed RNA using the GenePulser from BioRad with the following settings: 1.8 kV, 25 μF, 200 Ω. After electroporation, cells were seeded into a 75 cm^2^ tissue culture flask at a density of 1.0 × 10^5^ /mL. Six hours post electroporation, the medium was replaced by MEM 1% FCS, 1% L-glutamine and 0.5% neomycin. 24 h after electroporation, immunofluorescence staining was performed to monitor the efficiency of transfection. Figure S1 shows that no differences were observed for WT and mutants. 48 h post electroporation the supernatant containing the viral particles was harvested and clarified by centrifugation (10,000 rpm; 20 min; 4 °C: Beckmann JA 14).

### 2.6. Immunofluorescence (IF) Staining

Analysis of transfected as well as infected BHK-21 cells by immunofluorescence staining was carried out as described previously [26,28]. Cells were fixed on glass coverslips with acetone/methanol (1:1). Structural proteins were stained with a polyclonal rabbit anti-TBEV serum and the non-structural protein NS1 with a monoclonal mouse IgG (6E11, [29]) as primary antibodies. Alexa Fluor 448-conjugated goat anti-rabbit antibody (Thermo Fisher Scientific, Waltham, MA, USA) or rhodamine Red-X-conjugated goat anti-mouse antibody (Jackson ImmunoResearch Laboratories Inc., West Grove, PA, USA) were used as secondary antibodies. Hoechst dye served for the detection of cell nuclei.

### 2.7. Serial Passaging Experiments

Passaging of cell culture supernatants was performed as described in [30]. Briefly, BHK-21 cells were infected with supernatants containing WT and mutant viral particles harvested 48 h post electroporation. After incubation for one hour, the inoculum was replaced by MEM (1% FCS, 1% L-glutamine and 0.5% neomycin) and incubation was continued for six days at 37 °C. The supernatant of the first passage was harvested and used to infect fresh BHK-21 cells (passage 2). These steps were repeated until the fifth passage. Infectivity of the passaged viruses was monitored for the first, third and fifth passage by immunofluorescence staining.

### 2.8. Sequence Analysis

The cell culture supernatants of the transfected cells as well as of the first, third and fifth passages were harvested and incubated with 0.5% SDS, 0.8 µg proteinase K (Roche), 40 Units RNase inhibitor (Roche), 4 nM Tris(hydroxymethyl)-aminomethane (Tris) pH 8.0 and 2 nM ethylenediaminetetraacetate acid (EDTA) pH 8.0 for one hour at 37 °C. Viral RNA was isolated and purified by phenol-chloroform purification. cDNA transcription was performed using a cDNA synthesis kit (Roche) following the manufacturer´s protocol. cDNA was purified by phenol-chloroform extraction and was used for sequencing with the ABI Prism Big Dye Terminator Cycle Sequencing Kit (Applied Biosystems, Waltham, MA, USA) in combination with a capillary sequencer GA 3100 (Applied Biosystems) according to manufacturer´s instructions. The sequences were evaluated with the software Geneious Pro (version 5.0.4, Biomatters, San Diego, CA, USA).

### 2.9. Preparation of Cell Lysates

For quantification of intracellular E, cells were lysed by the addition of RIPA cell lysis buffer (1% Nonidet P-40 substitute, 0.5% sodium deoxycholate, and 0.1% sodium dodecyl sulfate; Thermo Fisher Scientific), supplemented with a mammalian protease inhibitor (Amresco Inc, Solon, OH, USA; under the VWR Life Science brand). For quantification of viral RNA by qPCR, cells were lysed by the addition of RLT buffer (Qiagen) supplemented with 1% ß-mercapto-ethanol.

### 2.10. ELISA for the Quantification of E Protein

The amount of E protein in cell lysates as well as cell culture supernatants was determined by a four-layer ELISA as described previously [31,32]. Briefly, microtiter plates (NuncU Maxisorp) were coated with a TBEV-specific polyclonal guinea pig serum (2.5 μg/mL) in carbonate coating buffer for 48 h at 4 °C. Samples and an internal TBEV standard (purified TBEV, [33]) were incubated in the presence of 0.4% sodium dodecyl sulphate (SDS) for 30 min at 65 °C [32]. Serial dilutions of the samples and standard were applied to the coated plates and incubated for 90 min at 37 °C. As primary antibody, a polyclonal rabbit anti-TBEV serum and as secondary antibody a peroxidase-conjugated donkey anti-rabbit immunoglobulin G (DAR-POX, GE Healthcare) were used. Protein concentrations were calculated with the Gen5 Data Analysis software using the standard curve.

### 2.11. qPCR for the Quantification of RNA Copies

RNA was isolated from cell lysates or supernatants with the RNeasy Clean up system (Qiagen) according to the manufacturer´s instructions. Isolated RNA was then quantified by reverse transcription qPCR as described previously [20,34,35]. Briefly, cDNA transcription was performed using the iScript cDNA synthesis kit (BioRad) according to the manufacturer´s instructions. Quantitative reverse transcription PCR was carried out with the TagMan Universal PCR master mix (Applied Biosystems).

### 2.12. Focus Assay

Focus assays were performed as described previously [26,35]. Briefly, a monolayer of BHK-21 cells was infected with cell culture supernatants or virus-containing peak fractions from sucrose density gradients. Two days after infection, cells were fixed with 4% paraformaldehyde, and foci were stained with a polyclonal rabbit anti-TBEV serum. As a secondary antibody, an alkaline phosphatase-labeled goat anti-mouse IgG (Sigma Aldrich) was used. SigmaFast Fast Red TR/naphthol AS-MX (Sigma Aldrich) served as substrate.

For determination of the stability of WT and mutant particles, the cell culture supernatant was diluted in MEM 5% FCS, 1% L-glutamine and 0.5% neomycin to 1.25 FFU/mL and incubated at 37 °C, 40 °C and 42 °C for 30 min prior infection. As a control, the WT particles were incubated at 4 °C.

### 2.13. ELISA for Determining the Maturation State of Viral Particles

The maturation state of WT and mutant viral particles was determined as described previously [26,32]. Microtiter plates (NuncU Maxisorp) were coated with a TBEV-specific polyclonal guinea pig serum (2.5 μg/mL) in carbonate coating buffer. After blocking with phosphate buffered saline (PBS) containing 1% bovine serum albumin (BSA), the viral particles (0.25 µg/mL) were added. Serial dilutions of monoclonal antibodies B4 (targeting E, [36,37]) or 8H1 (targeting prM, [29]) were added and detected via horseradish-peroxidase-conjugated rabbit anti-mouse antibody (Nordic Immunology, Susteren, The Netherlands). Mature and immature TBEV [32] were used as controls. For each sample the ratio of 8H1 to B4 of the area under the curve was calculated (GraphPad Software Inc., San Diego, CA, USA).

### 2.14. ELISA with Conformation-Sensitive Mabs

Microtiter plates (NuncU Maxisorp) were coated with a TBEV-specific polyclonal guinea pig serum (2.5 μg/mL) in carbonate coating buffer for 24 h at 4 °C. After blocking with phosphate buffered saline (PBS) containing 1% bovine serum albumin (BSA), the viral particles (0.5 µg/mL) were added for 1 h at 37 °C. After washing, mab IC3 (targeting DI) and mab A1 (targeting the FL and allowing measurement of its exposure) were added (0.5 µg/mL) and detected via horseradish-peroxidase-conjugated rabbit anti-mouse antibody (Nordic Immunology, Susteren, The Netherlands). To avoid particle destabilization, the assay was performed without detergent [35,38,39].

### 2.15. Ultracentrifugation (Pelleting) of Viral Particles

Harvested cell culture supernatants were applied to a 10% sucrose cushion in TAN buffer pH 8.0 (50 mM triethanolamine, 10 mM NaCl). Ultracentrifugation was carried out for 2 h at 50,000 rpm at 4 °C (Beckmann Ti 90) as described previously [26,40]. The resulting pellets containing the viral particles were resuspended in TAN buffer pH 8.0, supplemented with 0.1% bovine serum albumin. Quantification of E by a four-layer ELISA was carried out as described above.

### 2.16. Sedimentation Analyses

(a) Separation of SVPs and virions: WT and mutant pellets were subjected to rate zonal centrifugation using 5 to 30% sucrose gradients in TAN buffer pH 8.0. After centrifugation for 70 min at 38,000 rpm and 4 °C (SW40 rotor, Beckman), the gradients were fractionated and the amount of E protein per fraction was determined by a quantitative four-layer ELISA as described above.
(b) Separation of E dimers from E trimers: SVPs were incubated at low pH as described previously [25,41,42]. After back neutralization and solubilization with Triton X-100, sedimentation analysis was performed using 7% to 20% sucrose gradients in TAN buffer pH 8.0 containing 0.1% Triton X-100. After centrifugation for 20 h at 38,000 rpm and 15 °C (SW40 rotor, Beckman), the gradients were fractionated and the amount of E protein per fraction was determined by a quantitative four-layer ELISA as described above.

### 2.17. Statistics

Statistical significances were determined with GrapPad Prism 8 (GraphPad Software Inc., San Diego, CA, USA) by *t*-tests (two groups) or One-Way ANOVA followed by Tukey’s or Dunnett’s post-hoc tests (>two groups) using log-transformed values or square root transformed raw percentage data (not normalized to WT) to obtain normal distribution. *p* values < 0.05 were considered statistically significant.

### 2.18. GenBank Accession Numbers

Tick-borne encephalitis virus (TBEV, accession no. U27495), Omsk hemorrhagic fever virus (OHFV, accession no. AB507800), Powassan virus (POWV, accession no. AF310922), Karshi virus (KSIV, accession no. NC_006947.1), dengue virus 1 (DENV1, accession no. FJ687432), dengue virus 2 (DENV2, accession no. NC_001474), dengue virus 3 (DENV3, accession no. FJ850055), dengue virus 4 (DENV4, accession no. AY618990), Zika virus (ZIKV, accession no. KJ776791), Spondweni virus (SPOV, accession no. DQ859064), West Nile virus (WNV, accession number DQ211652), Japanese encephalitis virus (JEV, accession no. AF315119), Ntaya virus (NTAV, accession no. NC_018705), yellow fever virus (YFV, accession no. X03700), Wesselsbron virus (WSLV, accession no. DQ859058), Yokose virus (YOKV, accession no. AB114858), Entebbe bat virus (ENTV, accession no. DQ837641), Rio Bravo virus (RBV, accession no. JQ582840), Modoc virus (MODV, accession no. NC_003635).

## 3. Results

### 3.1. Secretion and Formation of Mutant Viral Particles

**Figure 2:**
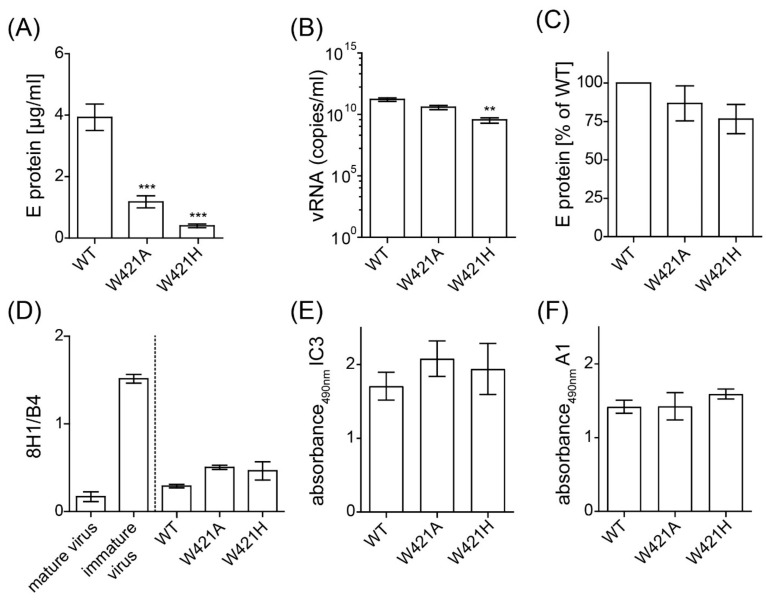
Quantification of E protein as well as viral RNA in the cell culture supernatants 48 h after transfection with WT and mutants and characterization of particle formation. (**A**) Concentration of E detected in the cell culture supernatant as determined by a quantitative ELISA. (**B**) Concentration of viral RNA in the cell culture supernatant as determined by qPCR. (**C**) Analysis of particulate nature of viral material in cell culture supernatants. Samples were subjected to ultracentrifugation and the amount of E in the pellet was determined by quantitative ELISA. Results are expressed as % E relative to WT. (**D**) Resuspended pellets were analyzed in ELISA with a prM-specific (8H1) and E-specific mab (B4) as described in Material and Methods. Immature virus and mature virus were used as controls. Results are expressed as the ratio of the area under the curve obtained with mab 8H1 relative to the one obtained with mab B4. (**E**,**F**) Resuspended pellets were analyzed in an ELISA with a DI-specific (IC3) and FL-specific mab (A1) as described in Material and Methods. Data are from at least three independent experiments; error bars represent the standard errors of the means (SEM). Asterisks indicate significant differences relative to the WT (ANOVA and Dunnett´s multiple comparison test; ** *p* < 0.01, *** *p* < 0.001).

To investigate the role of the strictly conserved W421 in the viral life cycle, we replaced this amino acid by the smaller and aliphatic alanine or by the charged histidine in an infectious clone of TBEV (W421A, W421H). In vitro-transcribed RNAs were transfected into BHK cells (see Materials and Methods), and the cell culture supernatants were harvested 48 h after electroporation. We analyzed the amount of secreted virus by quantifying E protein and viral RNA copies by ELISA and qPCR, respectively. The W421A mutant showed a significant reduction of E detectable in the cell culture supernatant (Figure 2A), but a significant difference was not detected by qPCR (Figure 2B). The W421H mutant was also significantly impaired with respect to the release of viral RNA (Figure 2A,B).

To determine the physical state of secreted E (soluble or particulate), we subjected the virus-containing cell culture supernatants to ultracentrifugation under conditions that allow the pelleting of subviral and viral particles (see Materials and Methods). The amount of E in the pellet and the supernatant were quantified by ELISA. Pelleting efficiencies of both mutants were not significantly different to the WT (Figure 2C).

To examine the maturation state of mutated virions released into the cellular supernatants, we analyzed the presence of prM in the pelleted particles by an ELISA with mabs specific for prM (8H1) and E (B4). As displayed in Figure 2D, the results obtained with the two mutants were not significantly different from the WT, indicating that furin cleavage of prM could occur properly in particles containing the mutations. The conformation of E at the surface of these particles was probed with the E-specific mabs IC3 (targeting DI) and A1 (targeting the FL in DII) in an ELISA (Figure 2E,F). Neither the binding of IC3 nor of A1 was altered by the mutations, indicating that E was properly folded.

### 3.2. Effect of Mutations on Virion Infectivity and Stability

**Figure 3:**
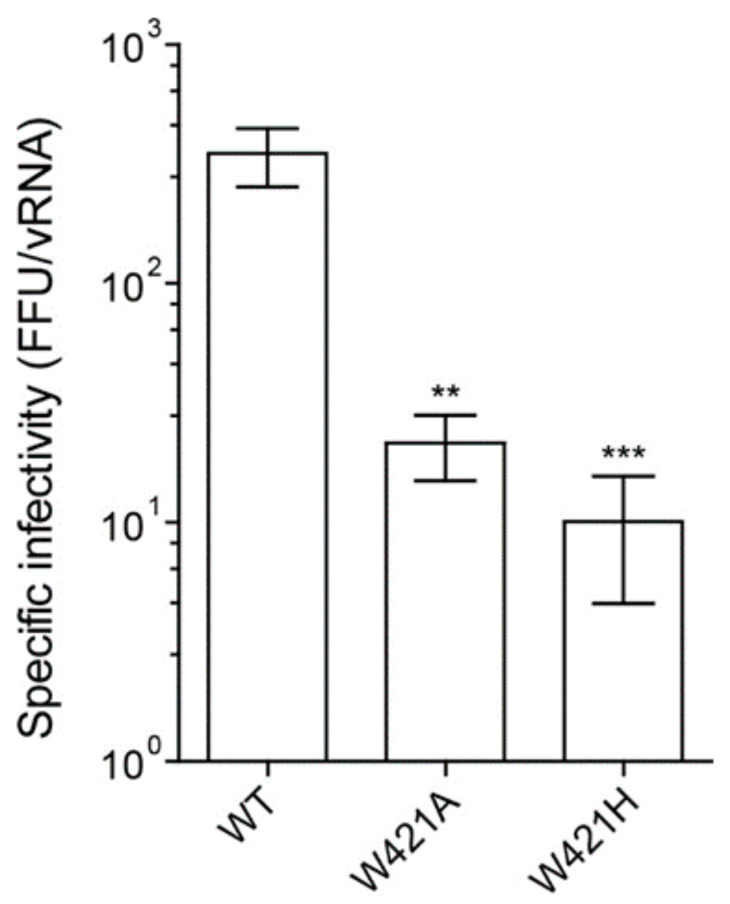
Infectivity of WT and stem mutants. Specific infectivities are indicated as number of focus forming units (FFU) per 10E7 RNA copies. Data are from at least three independent experiments; error bars represent the standard errors of the means (SEM). Asterisks indicate significant differences relative to the WT (ANOVA and Dunnett’s multiple comparison test ** *p* < 0.01, *** *p* < 0.001).

To evaluate the impact of W421 replacements on specific infectivities of mutated viruses, we determined focus forming units in the cell culture supernatants harvested 48 h after transfection and related those to viral RNA copy numbers. As shown in Figure 3, both mutants had significantly decreased specific infectivities compared to WT. In addition, focus morphology was affected, with a significantly lower mean size for the mutants, indicating that their efficiency of cell to cell spread was impaired (Figure S2). Notably, the W421A mutant displayed a much more dispersed pattern of small and large foci than WT and the W421H mutant, as also shown by the different ratios of maximum to minimum focus sizes (Figure S2).

**Figure 4:**
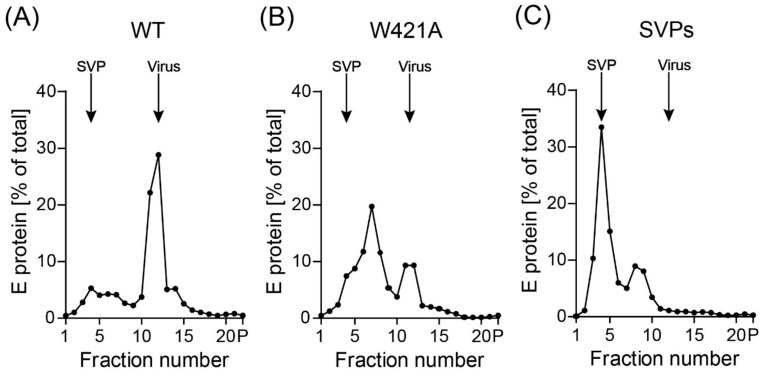
Rate zonal centrifugation of WT and mutant particles. Resuspended pellets (Figure 2C) containing viral as well as subviral particles (SVPs) were subjected to sedimentation analysis using continuous sucrose density gradients. The amount of E protein in each fraction was determined by ELISA. Sedimentation is from left to right. Representative examples out of two independent experiments are shown. The positions of SVPs and virions are indicated. (**A**) wildtype (WT), (**B**) W421A mutant, (**C**) SVPs.

Since the decreased infectivities of the two mutants might be caused by defects in specific viral functions and/or lower stability leading to disintegration of virus particles, we subjected WT and the W421A mutant to rate zonal density gradient centrifugation, which allowed a differentiation between particulate virus and possible breakdown products. The amount of the W421H mutant was too low for this type of experiment. In these analyses, we included non-infectious subviral particles (SVPs) as additional controls, because they are also formed naturally as a by-product of virus assembly [35,43], and the introduced mutations could favor the production of SVPs at the expense of virions. As shown in Figure 4A, WT particles sedimented predominantly as complete virions. For the W421A mutant, the virus peak was substantially smaller and a high amount of E was detected in the upper fractions of the gradient (Figure 4B). Since a defined SVP peak (compare with SVP control in Figure 4C) could not be identified, we conclude that E in the upper fractions most likely represents breakdown products of disintegrated particles.

### 3.3. Effect of Mutations on Fusion-Related Processes

The W421 mutations might reduce infectivity not only by influencing particle stability, as demonstrated above, but also by impairing the structural transitions required for membrane fusion. We therefore analyzed the low-pH-induced dimer-trimer transition of E, which is required for mediating membrane fusion [2]. For this purpose, we used non-infectious SVPs, which consist only of the two surface proteins anchored in a membrane and are a proven model to study entry-associated processes with flaviviruses [25,41,42]. SVPs are produced by the co-expression of prM and E in mammalian cells and undergo the same assembly pathway and exocytic release as infectious virions [24]. Although release was reduced for the W421A and W421H mutants (Figure S3A), the secreted material was particulate, like that of the WT (Figure S3B), and ultracentrifugation allowed the recovery of particles in sufficient amounts for further experiments.

**Figure 5:**
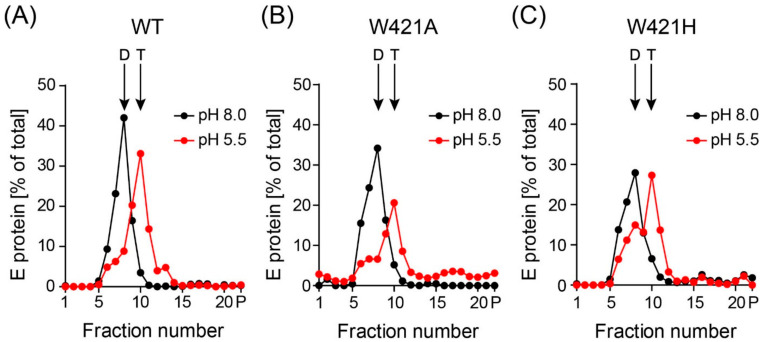
Sedimentation analysis of detergent-solubilized WT and mutant recombinant subviral particles (SVPs) after incubation at low pH. SVPs were incubated at pH 5.5 and pH 8.0. After back-neutralization, samples were solubilized and subjected to 7% to 20% sucrose density gradient centrifugation. The amount of E in each fraction was determined by a quantitative ELISA. Sedimentation direction is from left to right. Representative examples form three independent experiments are shown. (**A**) wildtype (WT), (**B**) W421A mutant, (**C**) W421H mutant. The position of E dimers (D) and trimers (T) are indicated by arrows.

To study the effect of mutations on E trimer formation at low pH, we incubated WT and mutant SVPs at pH 5.5 and subjected the samples to sedimentation analyses after solubilization, allowing the distinction between E dimers and trimers [25,41]. As shown in Figure 5, both mutants were still able to form trimers.

**Figure 6:**
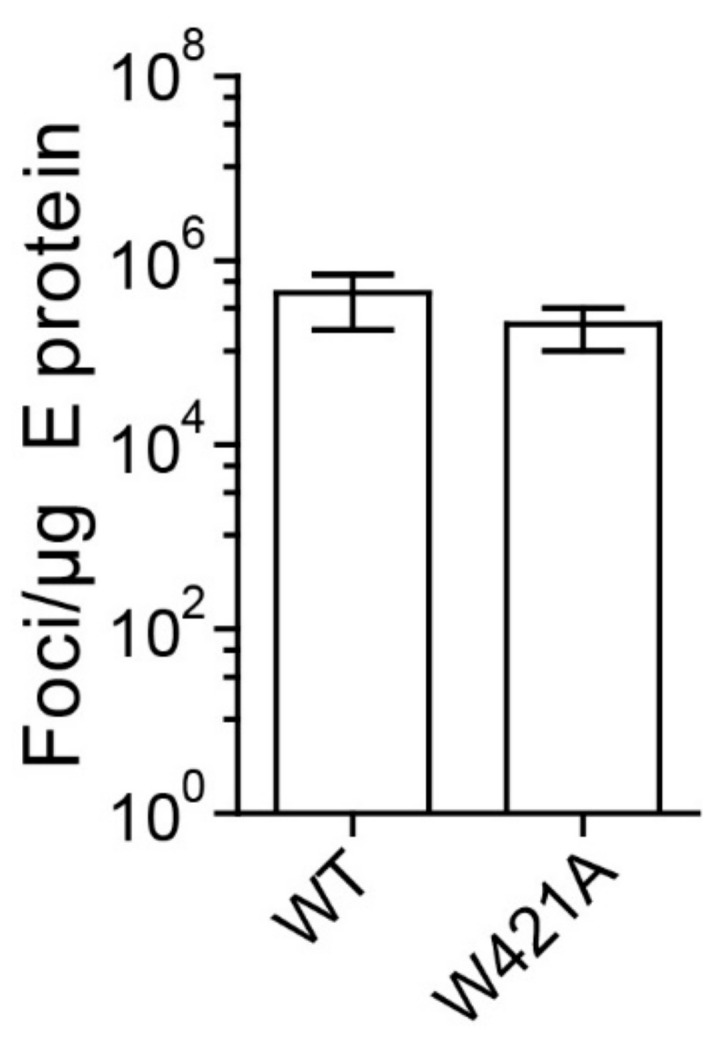
Specific infectivities of WT and W421A mutant virions form sucrose density gradients. Infectivities of virus peak fractions were determined in four independent focus assays. The infectivities are expressed as number of focus forming units (FFU) per µg E protein. Error bars represent the standard errors of the means (SEM). No significant difference to WT was observed (*t*-test).

This capacity is consistent with the finding that the W421A virus peak fractions from density gradients (Figure 4B) (i.e., that part of mutated particles that was not broken down by the concentration and centrifugation procedures) exhibited the same specific infectivities as WT when standardized to the amount of E found in these fractions (Figure 6). These data support the conclusion that the reduced infectivity observed with the mutants in cell culture supernatants (Figure 3) was primarily due to particle instability and/or improper formation of virus particles.

### 3.4. Rescue of Infectivity by Passaging Experiments

Since the mutations of W421 did not abolish infectivity completely, we attempted to gain additional insights from possible resuscitating mutations occurring during serial passaging experiments (Materials and Methods). As shown in Figure S4A, we observed an increased number of IF-positive cells with the W421H mutant from the third passage onwards. Sequencing of the viral RNA isolated from the cell culture supernatant of the corresponding passages revealed a compensatory mutation to leucine at the same site as the original mutation, and foci produced by this mutant had the same size as WT (Figure S4B). In contrast, passaging did not restore the infectivity of the W421A mutant, although it appeared less impaired than W421H.

**Figure 7:**
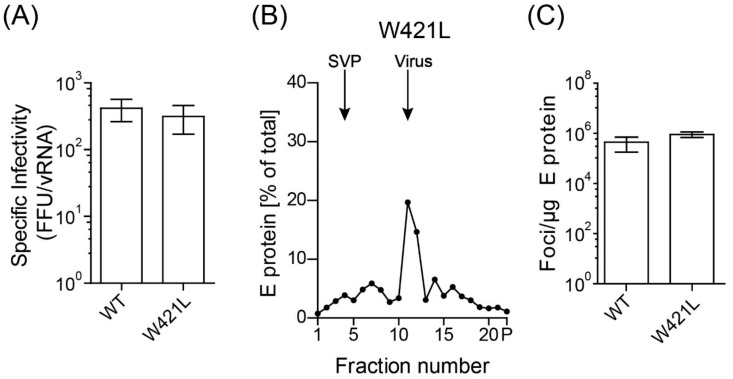
Infectious properties and particle state of W421L mutant. (**A**) Specific infectivities are indicated as number of focus forming units (FFU) per 10E7 RNA copies. (**B**) Resuspended pellets (Figure S4E) containing viral as well as subviral particles (SVPs) were subjected to sedimentation analysis using continuous sucrose gradients. The amount of E protein in each fraction was determined by ELISA. Sedimentation is from left to right. (**C**) Infectivities of virus peak fractions were determined in four independent focus assays and are expressed as number of focus forming units (FFU) per µg E protein. Data are from at least three independent experiments with mean values and error bars shown in red. Error bars represent the standard errors of the means (SEM). No significant difference to WT was observed (*t*-test).

To verify that the back-mutation to leucine was indeed responsible for the phenotypic resuscitation, we introduced this mutation into the infectious clone, and virus was produced by transfection of BHK cells with in vitro-transcribed RNA. Specific infectivity (Figure 7A) as well as the release of E and vRNA (Figure S4C,D) were restored to WT-like levels in the W421L mutant. As expected, particles were pelletable by ultracentrifugation (Figure S4E). Correspondingly, the infectivity of virus peak fractions from density gradients was identical to WT (Figure 7B,C), although slightly more slowly sedimenting E-containing material was detected (compare Figure 7B and Figure 4A).

Experiments with the same mutation engineered into SVPs showed that E carrying the W421L mutation was able to undergo the low-pH-induced dimer-to-trimer transition typical for the WT (Figure S4F).

Taken together, these data indicate that the leucine mutation resuscitated the defects caused by the W421A and W421H mutations, but appeared to be slightly less stable than the WT.

### 3.5. Effect of Mutations on Thermostability

The data so far point to an important role of W421 in the formation of stable virus particles. To expand further on this finding, we carried out thermal stability experiments. For this purpose, cell culture supernatants of the WT and the three mutants (harvested 48 h after transfection) were pre-incubated for 30 min at 37 °C, 40 °C and 42 °C before being subjected to focus assays. The number of foci obtained after incubation at the different temperatures were related to the number of foci of the controls pre-incubated at 4 °C.

**Figure 8:**
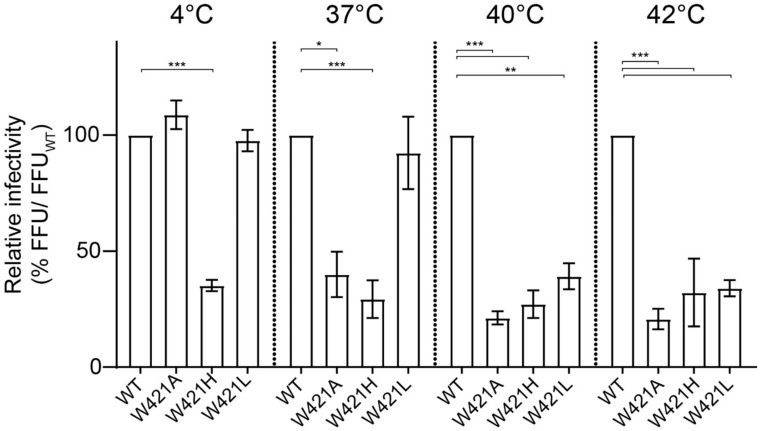
Thermal stability of WT and mutants. Relative infectivity is expressed as percent of focus forming units (FFU) of the WT and mutants at 37 °C, 40 °C and 42 °C relative to the FFU of the WT control at 4 °C. Data are from at least three independent experiments; error bars represent the standard errors of the means (SEM). Asterisks indicate significant differences relative to the WT at the corresponding temperature (ANOVA and Dunnett’s multiple comparison test; * *p* < 0.05, ** *p* < 0.01, *** *p* < 0.001).

As can be seen in Figure 8, the W421A and W421H mutants exhibited a significant decay of infectivity at all temperatures tested in comparison to WT (Figure 8). In contrast, the W421L mutant had the same stability as WT at 37 °C, and showed a significant reduction only at 40 and 42 °C (Figure 8). These data corroborate an important role of W421 in conferring stability to virus particles.

## 4. Discussion

In this study, we investigated the role of the strictly conserved hydrophobic residue W421 in the CS element of the E-stem in the viral life cycle. Replacement of this residue by alanine or histidine impaired the secretion (and probably formation) of viral particles but had no apparent effect on their maturation, which involves acidic-pH-induced conformational changes and oligomeric reorganizations of prM and E to allow furin cleavage of prM in the TGN [16]. Likewise, the mutants were still capable of displaying those acidic-pH-triggered conformational changes that convert metastable E dimers into stable trimers and are necessary for membrane fusion during virus entry. These data are consistent with experiments that showed that the stem region is not involved in the stabilization of the E-trimer [13] and a study with a W421I SVP mutant of TBEV that was not only able to form stable post-fusion trimers, but also to fuse efficiently with liposomes [25].

**Figure 9:**
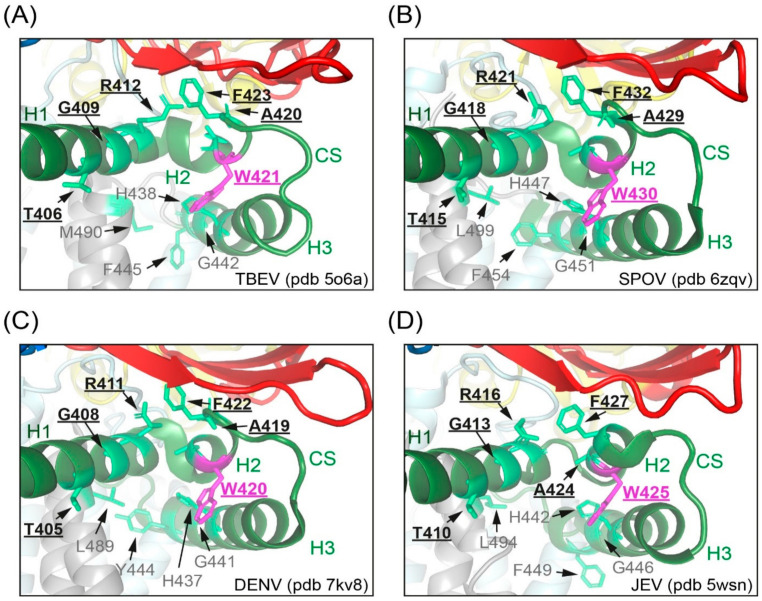
Stem residue W421 in the CS of TBEV and mosquito-borne flaviviruses. Close-up views of sites identified in Spondweni and dengue viruses as lipid-binding pockets [11,12] in mature TBEV (**A**), Spondweni virus (**B**), dengue virus serotype 2 (**C**), and Japanese encephalitis virus (**D**). Color code: E protein domain I, red; E protein stem, green; transmembrane (TM) domains, gray. W in the CS is shown in magenta and residues involved in lipid binding are shown in light green, respectively. Residues of lipid-binding site 1 are underlined and in bold, those of site 2 in grey [11,12].The structures were generated with PyMol (https://pymol.org, accessed on 5 July 2021) using the cryo-EM data of pdb files 5o6a [9], 6zqv [11], 7kv8 [12] and 5wsn [8]. TBEV, tick-borne encephalitis virus; SPOV, Spondweni virus; DENV, dengue virus; JEV, Japanese encephalitis virus.

The evidence presented in this work suggests that the dramatically reduced specific infectivities of supernatants from mutant-infected cells are related to the strongly reduced stability of viral particles, resulting in a tendency for disintegration and reduced resistance to thermal inactivation. Our findings are in agreement with the loss of infectivity of dengue virus serotype 2 particles with a W420A mutation (W421A in TBEV) [12], although detailed experiments for identifying the reasons of this defect were not performed with the dengue mutant. As revealed by cryo-electron microscopy (EM) structure analyses, CS connects two helices of the stem as a loop, both in mature and immature particles [3,4,5,6,7,8,9,10,11,12,44]. In mosquito-borne flaviviruses, W421 constitutes the last residue of H2, whereas in TBEV it is the first residue of the loop (Figure 1B and Figure 9). Studies resolving the structures of mature Zika, Spondweni and dengue serotype 2 viruses identified several residues (including W421, TBEV numbering) in the stem region of E as being part of lipid-binding pockets (Figure 9B,C; [10,11,12]). Comparison of the Spondweni and dengue structures with the structures of TBEV (Figure 9A) [9] and l as JEV (Figure 9D) [8] reveals similar orientations of the corresponding residues, suggesting that the tryptophan is conserved in all flaviviruses, because it mediates essential stem–stem as well as stem–lipid interactions, speculated to be important for stabilizing the mature flavivirus assembly [10,11,12]. Hardy et al. [12] suggested that the two lipid pocket factors are recruited after prM cleavage in the TGN, which is consistent with our finding that the mutants had no apparent defect in virus maturation (Figure 2D). Notably, a lipid-binding pocket is also formed by the membrane-associated helices of the envelope glycoproteins E1 and E2 on the surface of mature alphaviruses, with a tryptophan (conserved among mosquito-borne alphaviruses) being involved in the stabilization of this site [45].

As shown by passaging experiments, WT properties could be regained by a same-site mutation to leucine which, via its branched side chain and hydrophobic properties, seems to be able to provide interactions sufficient for the formation of functional infectious viruses. Nevertheless, although similarly stable at 37 °C, W421L particles exhibited reduced infectivity after incubation at 40 °C and 42 °C (Figure 9), suggesting a somewhat weaker capacity to support the network required for stem–stem interactions and pocket factor binding. The fast appearance of the leucine mutation in our passaging experiments might be due to the requirement of a single nucleotide change in the W421H mutant to generate leucine (Table S1). In the case of the W421A mutant, however, for which infectivity could not be rescued, at least two nucleotide substitutions would have been necessary for a corresponding compensatory mutation or reversion to WT.

Since flaviviruses assemble first in an immature form, the mutations could already have an impact on the formation of immature particles in the ER. A structure of immature TBEV is not available so far, but high-resolution prM-E structures of mosquito-borne dengue serotype 1 and Spondweni viruses [4,11] and the insect-specific Binjari virus [44] show that the lipid-binding pockets are absent and that the transmembrane domains and the stem are more tightly packed [11]. Mutating the conserved tryptophan might impair these interactions, leading to inefficient assembly and/or intracellular budding of immature particles, similar to what has been observed with the structurally related E1 protein of Semliki forest virus (SFV). Several mutations in the stem region of SFV E1 affected budding and growth of virus particles [46]. A reduced release of viral and subviral particles was also seen with helix-breaking mutations in the stem of dengue virus serotype 2 [47].

Our study contributes to a better understanding of the functional role of the CS element in the stem of flavivirus E proteins. Nevertheless, important questions remain open and can be topics of future research. These include investigations on the role of the stem in the assembly of immature particles, the precise stage in the viral life cycle when lipid pocket factors are acquired and the atomic details of interactions between lipids and the corresponding residues in the stem. In-depth knowledge of these parameters can make the pocket regions valuable targets for developing antiviral agents.

## Figures and Tables

**Table 1:** Primers used in the first PCR to amplify the “megaprimers” for the second PCR.

Primer	Sequence (5′to 3′)
rf ^1^ stem fwd ^2^	GAG TCA TCA ATG GTT CCA AAA AGG GAG CAG CAT CGG AAG GGT TTT CC
rf stem rev ^3^	GGC GCT TTC AAC AGC ATC TTC GGG GGA GTG GGG TTT CTA C

^1^ rf, restriction free; ^2^ fwd, forward; ^3^ rev, reverse.

**Table 2:** Primers used for site-directed mutagenesis of the pSV-PE WT vector containing prM and E of TBEV Strain Neudoerfl.

Mutation	Primer	Sequence (5′to 3′)
W421A	fwd ^1^	GTG ATA GGA GAG CAC GCC GCA GAC TTC GGT TCT GCT GGA
W421A	rev ^2^	TCC AGC AGA ACC GAA GTC TGC GGC GTG CTC TCC TAT CAC
W421H	fwd	ATA GGA GAG CAC GCC CAT GAC TTC GGT TCT GCT
W421H	rev	AGC AGA ACC GAA GTC ATG GGC GTG CTC TCC TAT
W421L	fwd	ATA GGA GAG CAC GCC CTT GAC TTC GGT TCT GCT
W421L	rev	AGC AGA ACC GAA GTC AAG GGC GTG CTC TCC TAT

^1^ fwd, forward; ^2^ rev, reverse.

## Data Availability

Data is contained within the article or supplementary material.

## Supplementary Materials

The following are available online at https://www.mdpi.com/article/10.3390/v13091727/s1, Table S1: Codons at position 421 of the E protein for WT and mutants, Figure S1: Intracellular expression of viral proteins and quantification of E and viral RNA in transfected cell lysates 6, 18, 30, 42 and 54 h post electroporation (pE), Figure S2: Focus Morphology of WT and stem mutants, Figure S3: Characterization of SVP productions, Figure S4: Characterization of the engineered W421L mutant.
