# Supplementary material for: An Absolutely Conserved Tryptophan in the Stem of the Envelope Protein E of Flaviviruses Is Essential for the Formation of Stable Particles
Source: Viruses. 2021 Aug 30;13(9):1727. doi: 10.3390/v13091727 (PMC8473212; doi:10.3390/v13091727)
Supplement: Supplementary file 1 [file viruses-13-01727-s001.zip › viruses-1350431-supplementary.pdf]

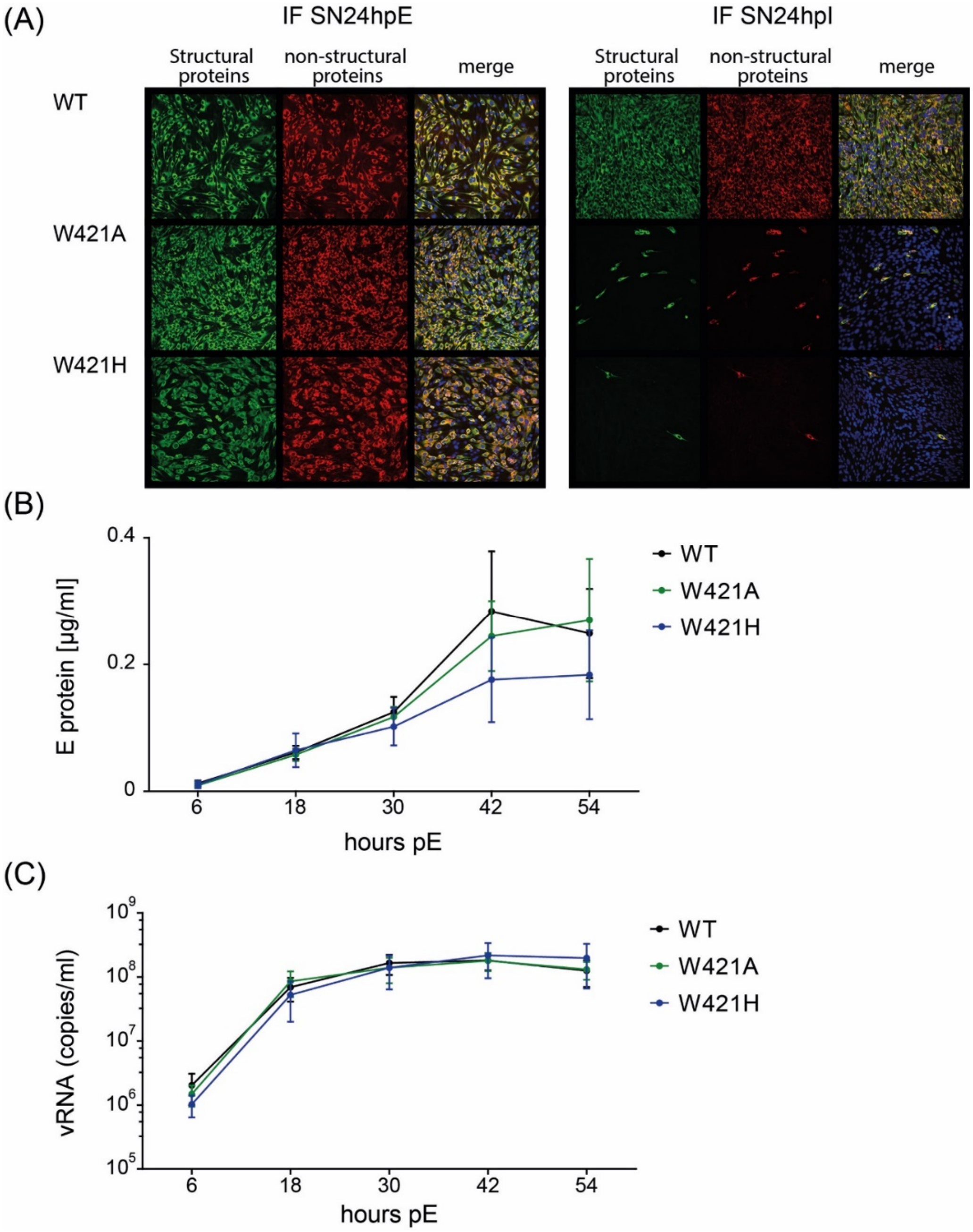


**Figure S1: Intracellular expression of viral proteins and quantification of E and viral RNA in transfected cell lysates 6, 18, 30, 42 and 54 hours post electroporation (pE).**

(A) Immunofluorescence staining 24 hours post electroporation (left panels) and post infection (right panels) of WT and stem mutants W421A and W421H. Staining was carried out with a polyclonal serum recognizing TBEV prM/M and E (green fluorescence) and a monoclonal antibody recognizing NS1 (red fluorescence). Nuclear DNA was stained with Hoechst (blue fluorescence). Representative examples of at least three independent experiments are shown. (B) Quantitative ELISA and (C) qPCR of cell lysates. Data are from at least three independent experiments; error bars represent the standard errors of the means (SEM). No significant difference to WT was observed (ANOVA and Dunnett's multiple-comparison test).

(A)

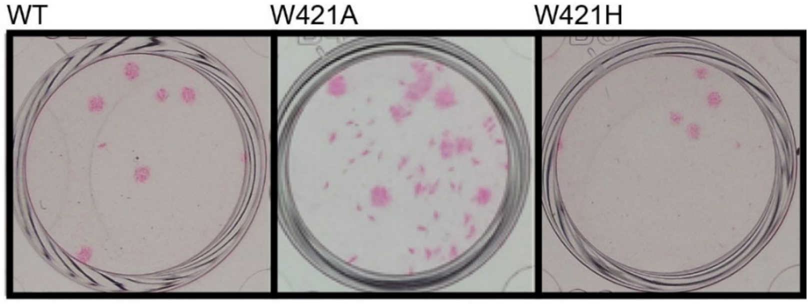


(B)

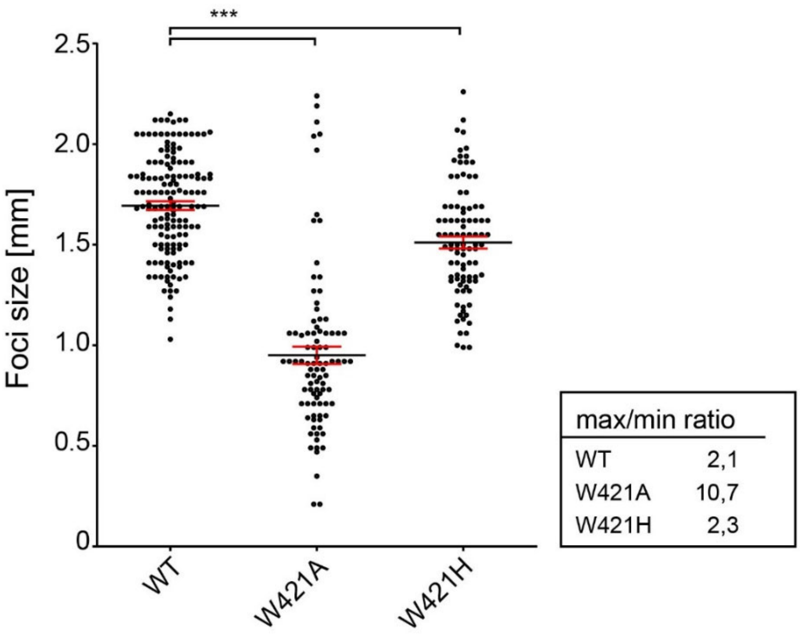


**Figure S2: Focus Morphology of WT and stem mutants.**
(A) Representative photographs of focus assays. (B) Distribution of focus sizes, determined for 100 foci-of WT and mutant particles. Data are from at least three independent experiments. From each single experiment, sizes of clearly separated foci were determined. The inset shows the maximum (max) to minimum (min) ratios of focus sizes. Error bars represent the standard errors of the means (SEM). Asterisks indicate significant differences relative to the WT (ANOVA and Dunnett's multiple comparison test; *p<0.05, **p<0.01, ***p<0.001).

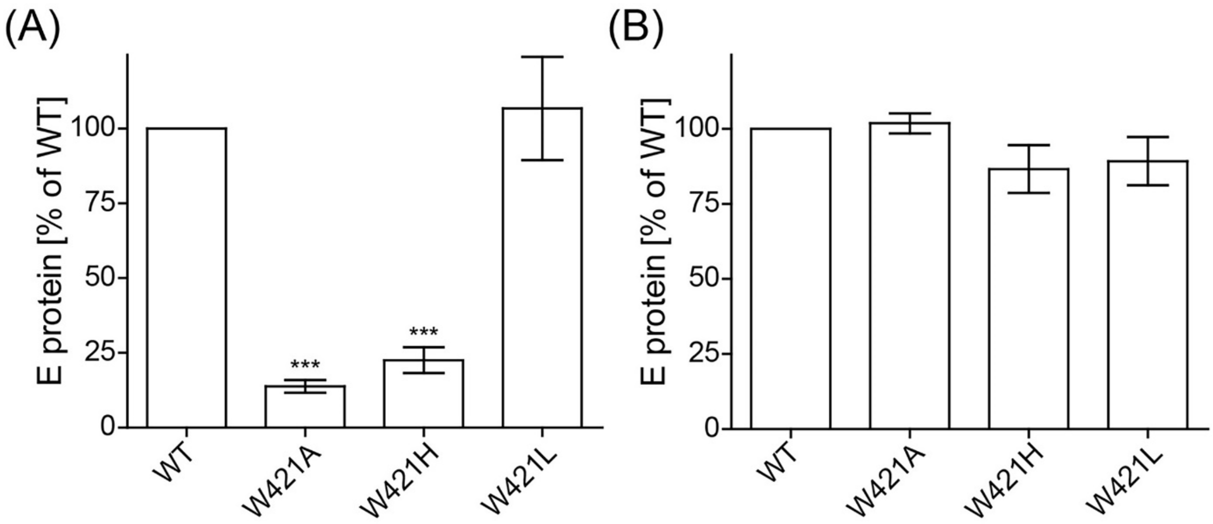


**Figure S3: Characterization of SVP productions**

(A) Amount of E released into transfected cell culture supernatants as determined by a quantitative ELISA. (B) Pelleting efficiency. Cell culture supernatants were subjected to ultracentrifugation and the amount of E was determined by a quantitative ELISA. Results are expressed as % E protein relative to WT. Data are from at least three independent experiments; error bars represent the standard errors of the means (SEM). Asterisks indicate significant differences relative to the WT (ANOVA and Dunnett´s multiple comparison test).

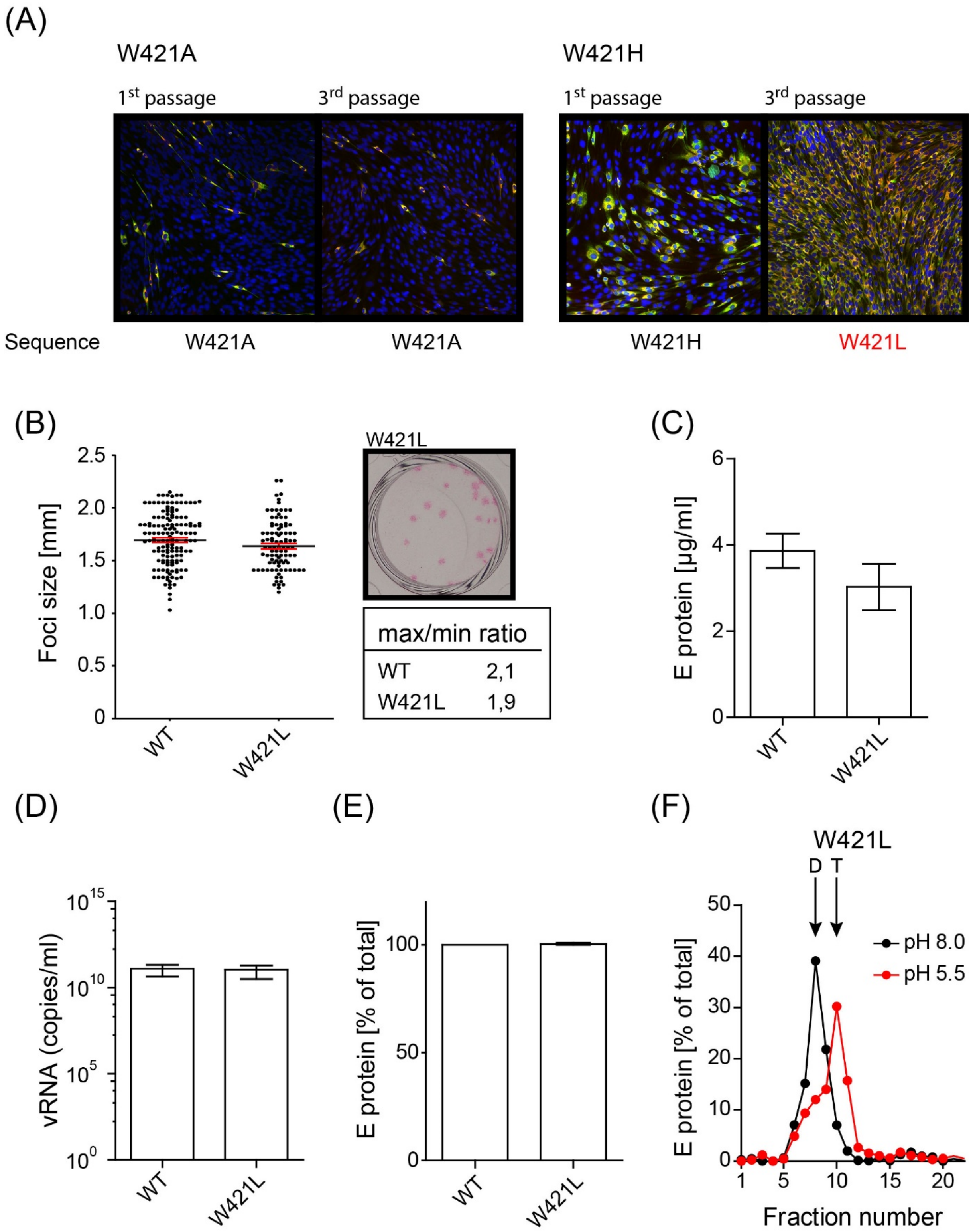


**Figure S4: Characterization of the engineered W421L mutant**

(A) Immunofluorescence staining 24h post infection using the cell culture supernatant of the 1st and 3rd passages of stem mutants W421A and W421H. Staining was carried out with a polyclonal serum recognizing TBEV prM/M and E (green fluorescence) and a monoclonal antibody recognizing NS1 (red fluorescence). Nuclear DNA was stained with Hoechst (blue fluorescence). Representative examples of at least three independent experiments are shown. (B) Morphology of foci. In total, sizes of 100 foci for W421L particles were

determined (left). Representative photograph of focus assays (right). From each single experiment, sizes of clearly separated foci were determined. The inset shows the maximum (max) to minimum (min) ratios of focus sizes. (C, D) Release of E and RNA into the cell culture supernatant determined by ELISA (C) and qPCR (D), respectively. (E) Pelleting efficiency. Cell culture supernatants were subjected to ultracentrifugation and the amount of E was determined by quantitative ELISA. Results are expressed as % E protein relative to WT detected in the pellet. (F) Sedimentation analysis of detergent-solubilized W421L recombinant subviral particles (SVPs) after incubation at low pH. SVPs were incubated at pH 5.5 and pH 8.0. After back-neutralization, samples were solubilized and subjected to 7 to 20% sucrose density gradient centrifugation. The amount of E in each fraction was determined by a quantitative ELISA. Sedimentation direction is from left to right. Representative examples from three independent experiments are shown.

**Table S1:** Codons at position 421 of the E protein for WT and mutants

| Virus | Amino Acid | Codon |
|---|---|---|
| WT | Tryptophan | UGG |
| W421H | Histidine | CAU |
| W421A | Alanine | GCA |
| W421L | Leucine | CUU |
